# The duality of the BBB: breaking the myth of the blood-brain barrier breakdown

**DOI:** 10.1186/s12987-025-00732-y

**Published:** 2025-11-27

**Authors:** Frédéric Calon

**Affiliations:** 1 Faculté de pharmacie, Université Laval, Québec, Canada; 2 Axe Neurosciences, Centre de recherche du CHU de Québec, Université Laval, 2705, Boulevard Laurier, Room T2-67, Québec, QC, G1V 4G2 Canada

## Abstract

Research on the blood-brain barrier (BBB) has greatly evolved over the past 20 years, with growing recognition of its role as a multicellular complex regulating brain homeostasis. Previously confined to pharmaceutical sciences, the BBB has now become a growing focus of interest for neuroscientists and clinicians. However, the word ‘barrier’, implying something that can be broken, opened, or disrupted, can lead to confusion when one tries to relate this concept to its underlying cell biology. Here, echoing the fundamental question posed by Lina Stern when she first defined the BBB in 1921, I suggest that the confusion stems from conflating the physicochemical properties intrinsic to the barrier with the living biological multicellular interface. Notwithstanding its complexity, the BBB is now often simplistically portrayed as “permeable”, particularly in the context of prevalent diseases, such as Alzheimer’s, depression, multiple sclerosis, or stroke. Such overly simplified concepts promoted over the past two decades have led to misconceptions that hinder a proper understanding of the BBB, affecting both the general public and seasoned scientists. This misunderstanding is not without harmful clinical impact as many interpret the BBB as something that often breaks, leading to a massive entry of drugs and other blood-borne compounds into the brain, which is very rarely the case. After outlining the likely causes of these misconceptions and trying to define the concepts of “BBB permeability” and “brain bioavailability”, I offer several recommendations: (1) more frequent use of quantitative methods involving small hydrophilic compounds to measure BBB integrity; (2) avoid terms such as ‘BBB disruption,’ ‘opening,’ or ‘breakdown,’ and instead favor terms like ‘dysfunction’ or, where appropriate, ‘leakage’, essentially when describing biological defects assessed by changes in large molecule localization; and (3) always account for the dual nature of the BBB, as both a physicochemical barrier and a living biological interface.

## Introduction

Shortly after the First World War, Dr Lina Stern in Switzerland was probably the first scientist to use the term “*barrière hémato-encéphalique*” in French, translated as the blood-brain barrier (BBB) in English. After testing a large number of compounds in animals, Dr. Stern then posed this question about the BBB in 1921: **is it a biological or vital phenomenon**,** or a physical or physicochemical phenomenon?** [translated from *S’agit-il d’un phénomène biologique ou vital*,* ou bien d’un phénomène physique ou physico-chimique*] [1–3].

**Fig. 1:**
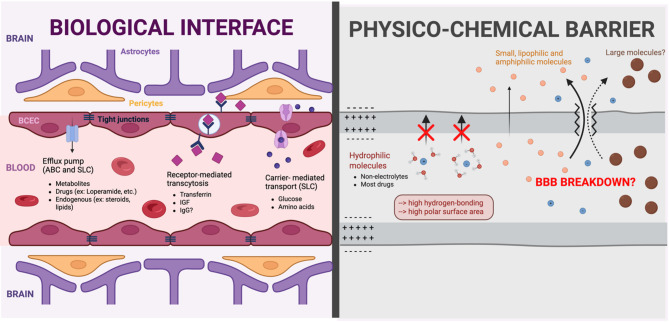
The dual nature of the blood-brain barrier (BBB). The BBB operates as both a dynamic biological interface (left) and a selective physicochemical barrier (right). Endothelial cells, pericytes and astrocytic end-feet forming the BBB regulate molecular exchange through efflux pumps, receptor-mediated transcytosis and carrier-mediated transport. The intrinsic physicochemical properties of the BBB strictly limit the entry of hydrophilic molecules, particularly with a high hydrogen-bonding potential, while allowing passive diffusion of small lipophilic or amphiphilic compounds. Claims of BBB disruption or breakdown are most often inferred from qualitative or semi-quantitative microscopic assessments using large tracer molecules. Yet, in such circumstances, small hydrophilic compounds should also penetrate the brain, although they are rarely evaluated with quantitative methods. In sum, any investigation of the BBB, whether focused on its “permeability” or its role in health and disease, should recognize its dual nature, encompassing both a physicochemical barrier and a dynamic biological interface. BCEC, brain capillary endothelial cells; IGF, insulin-like growth factor; IgG, immunoglobulin G; ABC, ATP-Binding Cassette (efflux); SLC, solute carrier (influx/efflux)

One hundred years later, this question remains at the heart of the understanding (or misunderstanding) of the BBB. The physicochemical view prevailed until the 2000s, whereas the biological view has since gained precedence. However, these two aspects of the BBB are not captured or measured in the same way experimentally, nor do they have the same consequences on the pathophysiology of CNS diseases. The conclusions drawn from a biological experiment does not necessarily lead to a clear interpretation at the physicochemical level and vice versa. I argue that this confusion arises from the dual roles of the BBB (Fig. 1), which are the root of many current misinterpretations surrounding the BBB.

## What is the BBB? Biological and physicochemical viewpoints

Common contemporary definitions of the BBB typically begin with a description of the cerebral microvasculature, highlighting its biological and histological properties, as well as its array of transporters [4–7, 8]. This perspective emphasizes a biological view of the BBB, framed within the integrative concept of the neurovascular unit (NVU) [9]. Earlier reports rather defined the BBB based on results from pharmacological distribution experiments [5, 10, 11]. This physicochemical view arose from the inability of many systemically administered compounds to reach the CNS. Both standpoints assume that the vascular network in the brain exhibits sets of properties that are specific and not found in other organs. Both views are compatible, and there is clearly a biological basis for the physicochemical characteristics of the BBB – and vice versa.

In the initial experiments conducted more than 100 years ago by various scientists, including Edwin Goldmann, Paul Ehrlich and Lina Stern [2, 3, 12–15], it was noted that dyes administered intravenously did not enter the CNS, leaving the brain “white as snow”, while distributing readily throughout the rest of the body. It was initially and logically assumed that this resulted from the absence of a putative binding site in the CNS. However, Goldmann and Stern subsequently showed that injecting the dye directly into the cerebrospinal fluid (CSF) of animals led to complete staining of the brain and spinal cord, while the rest of the body remained unstained. The existence of a “barrier” between the blood and brain of mammals was henceforth demonstrated.

The dramatic growth in pharmaceutical product development from the 1930s to the early 2000s highlighted the BBB as a major obstacle in CNS drug discovery. Drugs that successfully received approval for CNS indications were predominantly small molecules, such as antipsychotics and antiepileptics. From that period onward, assessing the ability of a molecule to cross the BBB became a critical determinant of its developmental potential. Subsequently, a few research teams strongly emphasized this limitation and initiated research efforts to enhance the molecular transport of novel drugs across the BBB [5, 16].

The gradual shift toward a biological view of the BBB brought significant new insights into the cellular composition of the cerebrovascular network. Respective key roles of endothelial cells, pericytes, astrocytic end-feet, and the extracellular matrix were uncovered [7, 8]. This cellular perspective also revealed the dynamic nature of the BBB, both in preserving CNS homeostasis and in its involvement in pathological conditions. Unfortunately, as the physicochemical view began to fade, it fostered bold claims suggesting that cellular changes could result in dramatic disruption or “breakdown” of the structure or function of the BBB.

Although both views of the BBB are fundamentally correct, the favored perspective will profoundly alter not only its perceived role, but also the interpretation of experimental results aimed at studying it. However, a severe dysfunction of the BBB can occur without change in permeability, and conversely, the physicochemical barrier function may weaken without massive biological alterations. For example, a dysfunction in endothelial cells could impair the uptake of energy substrates like glucose, without impacting drug biodistribution. On the other hand, perturbing the BBB with an osmotic shock or low-intensity ultrasound may transiently facilitate drug entry into the brain parenchyma without significantly affecting other biological functions of endothelial cells, such as efflux mechanisms [5, 17–19]. In other words, changes in specific cellular functions of the BBB do not necessarily imply alterations in its basic physicochemical properties, just as transient changes in physicochemical properties may not reflect widespread underlying cellular changes.

These two views of the BBB also imply distinct timeframes, particularly regarding the transport of compounds across its interface (Fig. 1). The physicochemical gating of the BBB governs molecular transport on extremely short timescales, typically ranging from milliseconds to minutes. In contrast, biological transport mechanisms, such as receptor-mediated transcytosis and CSF-perivascular pathways, can operate over longer durations, from several minutes to hours or even days [4, 20–25]. Consequently, rapid experimental approaches, such as in situ brain perfusion [26], are particularly well-suited for assessing physicochemical properties but may not adequately capture slower modes of transport that rely on extended circulation times and high plasma exposure (high areas under the curve). Conversely, methods evaluating brain concentrations after prolonged circulation periods may overestimate the true rate of BBB transport, as they also reflect systemic exposure and accumulation via other slower translocation mechanisms.

## How to assess BBB ‘permeability’?

Permeability is one of these BBB-related concepts whose interpretation is critically affected by this duality. In a physical context, permeability typically refers to the ability of a material to allow fluids (such as water, oil, or gas) to pass through it. A key point to note is that early experiments giving rise to the concept of the BBB involved relatively small hydrophilic dyes, such as trypan blue (873 g/mol), which readily distributed to peripheral organs but not to the brain when administered systemically, and conversely could not escape the CNS when injected into the CSF [12]. Subsequent studies have reported many other compounds with the same striking inability to penetrate the CNS, including histamine (111 g/mol), curare alkaloids (~ 680 g/mol) and clarithromycin (748 g/mol), to name just a few [3, 5]. Lisa Stern, in particular, investigated a large series of small compounds, either injected systemically or directly in the CSF [1–3]. In the second half of the 20th century, pharmaceutical companies routinely conducted similar experiments with radiolabeled molecules in early-stage drug development. Although molecular size and hydrophilicity are critical, high hydrogen bonding potency (donors/acceptors) appears as the main physicochemical roadblock to BBB crossing [27–29]. This observation is likely attributable to the formation of extensive bonding networks with water molecules, which increase the molecule’s effective polar surface area and may rigidify its conformation, thereby hindering its diffusion across the tight junctions of endothelial cells of the BBB [28, 29].

In summary, what was learned then and remains true today, is that the unique feature of BBB cells is their specific ability to fully restrict passage of small, hydrophilic molecules with high hydrogen bonding potential. Consequently, measures of BBB permeability should be performed using such molecules. Unfortunately, claims of increased BBB permeability or BBB breakdown are rarely performed with compounds that truly show a dichotomous distribution (absence in the brain but presence in peripheral organs after systemic administration). Due often to logistical reasons, larger, more complex compounds with their own advantages (e.g. detectable in microscopy) are more commonly used. A quantitative technique such as radiolabeling is thus being increasingly replaced by fluorolabeling, which attaches another molecule that can alter the intrinsic properties of the studied compound or vascular marker. Routinely, an increase in the overall quantity of a large fluorescent molecule recovered in brain samples following peripheral administration is often interpreted as a barrier breach. One of the most commonly used markers is the fluorescent dye Evans Blue (EB), which is relatively small (961 Da) and hydrosoluble. Its chemical structure is very similar to that of Trypan Blue, which was used in older studies or for cell viability assays, but Evans Blue can be more easily measured spectrophotometrically [5, 12, 30]. Yet, EB strongly binds to serum albumin, thereby forming a high molecular weight protein tracer in blood (69 kDa) when injected systemically [30]. This EB-albumin complex is indeed blocked by the BBB but also poorly distributed in many peripheral organs such as the heart [30–32]. Hence, the absence or presence of EB fluorescence in the CNS does not say anything specific about the BBB, compared to other blood-organ interfaces. In addition, following IV injection, the distribution of EB and its albumin-bound form is modulated by pharmacokinetic alterations, metabolism, and albumin levels. Simply put, this means that the localization of EB and of other large fluorolabeled molecules in the brain may result from various factors unrelated to actual changes in the physicochemical properties of the BBB.

Obviously, the BBB greatly limits the cerebral bioavailability of large molecules, including monoclonal antibodies (mAbs) and albumin. However, in this respect, the BBB is not fundamentally different from other blood–organ interfaces. For instance, immunoglobulins (IgGs) typically remain within circulation and do not penetrate most organs. Direct measurements of mAbs following peripheral administration show brain concentrations in the low nanomolar range, with levels in most other organs about one order of magnitude higher [3–35]. These differences are not as dramatic as with small nonelectrolytes that led to the discovery of the BBB. In other words, as molecule size increases, the distinction between the physicochemical barrier effect of the BBB and that of other organs becomes less pronounced. Thus, when it comes to large molecules, their transport across the BBB is governed less by physicochemical parameters and more by the biological activity of BBB cells. Furthermore, if the objective of an experiment is to assess the permeability of a molecule across the barrier, or to evaluate the intrinsic permeability of the barrier itself, the readout should be an actual quantitative measure of permeability. A typical BBB permeability coefficient can be expressed in terms of quantity over time, reflecting the rate at which a substance diffuses through a membrane. Assuming that the BBB surface area per unit of brain tissue is consistent throughout the brain, we can aggregate the weights, yielding units of quantity per time per weight (e.g. µmol·s⁻¹·g⁻¹). Caution should be taken when discussing changes in permeability without a clear quantitative assessment.

In sum, given the degree of disconnect between biological and physicochemical properties of the BBB, it is more accurate – except in specific quantitative experimental settings – to refer to BBB dysfunction or alteration rather than to a change in permeability. The latter term does not apply to the passage of cells or large molecules, but rather to that of small hydrosoluble compounds.

## BBB crossing and brain bioavailability

It is also crucial to distinguish between the concepts of BBB crossing and brain bioavailability. Some molecules may easily cross the BBB but have very low distribution in the brain [36]. An obvious example is cholesterol, which readily crosses the BBB, but has null CNS biodistribution due to its binding to large circulating entities [37, 38]. In addition, while highly lipophilic compounds readily cross the BBB, systemic administration leads to their rapid distribution across all tissues, which quickly reduces their AUC and, consequently, limits their brain bioavailability [33, 39]. Conversely, other molecules may cross the BBB at a very slow rate, but because they have prolonged exposure in circulation and high AUCs, they may still achieve sufficient concentrations in the brain interstitial fluid. IgGs (i.e. mAbs) are typical examples [34, 40, 41]. Indeed, they have a low brain-to-plasma ratio (< 0.01%), low percentage of the injected dose reaching the brain (< 0.01%), and low brain uptake rate (< 0.015 µl.g^− 1^.s^− 1^) [34, 42, 43]. The recent success of lecanemab and donenemab suggests target engagement, likely explained by their very strong affinity, which compensates for their CNS concentrations in the low nM range. Some large drugs may also access the CNS by crossing the blood-CSF barrier first and then moving through perivascular distribution pathways [20, 21, 36]. This is not to mention that drugs can also influence the CNS indirectly through peripheral mechanisms, such as cardiometabolic pathways or the gut–brain axis [44]. Ultimately, framing the BBB as either open or closed, or BBB crossing as a yes or no question, is not particularly useful when assessing the brain bioavailability of a drug and its potential to exert effects on the CNS.

## Is there such a thing as BBB breakdown?

The use of extreme terms like “breakdown” or “disruption,” frequently encountered in the scientific literature and at meetings [45], can have negative consequences. Such language is difficult to interpret as anything other than a widespread increase in BBB permeability across the brain. Unfortunately, these interpretations are often not grounded in accurate data but instead on experiments using large molecules and qualitative assessments. If the entry of large molecules into the brain were truly due to a breach of the BBB, then such a breach should allow even greater entry of smaller molecules (Fig. 1). In other words, if a widespread ‘opening’ of the BBB genuinely occurs, one would expect a substantial influx of small hydrophilic molecules – normally excluded from the brain due to their physicochemical properties – even more so than large molecules.

## What would be the clinical consequence of a BBB breakdown?

The brain is a highly irrigated organ. It is estimated that 1000 L of blood flows through the human brain each day. The blood is replete with small and large compounds that have limited access to the brain parenchyma. If a BBB breakdown were to truly occur, there would be consequences. Let’s explore a few.

Over the course of evolution, many physiological processes – such as those involving lipids, the immune system, and monoamine transmission – have become compartmentalized between the periphery and the CNS. Catecholamines (e.g., dopamine, norepinephrine) and indolamines (e.g., serotonin) are monoamine neurotransmitters synthesized both in the periphery and in the brain, but their central and peripheral pools are functionally and anatomically separated by the BBB, which prevents exchanges. Catecholamines often rise to mid-nanomolar range in the human blood (i.e. up to 50 nM for epinephrine, and up to 2 nM for dopamine, after intense exercise) [46–48]. Owing to the presence of hydroxyl moieties in their structure, peripherally produced catecholamines are unable to cross the BBB. A true ‘BBB breakdown’ would expose the brain to blood-borne catecholamines, potentially mimicking the effect of stimulants such as amphetamines. In such a scenario, even mild physical exercise could trigger a noticeable euphoric response.

It is well known that the BBB remains a critical limitation to brain drug delivery. Nearly 69% of the US population has used at least one prescription medication in the past 30 days [49]. Yet, the majority of commonly prescribed drugs do not cross the BBB, mainly because of their physicochemical characteristics and/or the intervention of efflux pumps like P-glycoprotein (P-gp) transporters [5]. This is generally beneficial as the BBB offers protection against unwanted CNS adverse effects. In this context, the integrity of the BBB is a key determinant of systemic drug safety. Were the BBB to undergo a functional disruption or structural breakdown in common diseases, it would lead to increased CNS exposure to peripheral drugs, resulting in neurotoxicity, altered therapeutic responses, and a higher incidence of adverse effects. If such cases of disruption were both common and clinically significant, they would be well-documented.

Let’s consider age-related neurodegenerative diseases, which affects tens of millions of people worldwide. Although there is strong experimental evidence of barrier dysfunction in these chronic diseases [50–53], such findings are often interpreted as proof of BBB disruption, frequently inferred from DCE-MRI data or cerebrospinal fluid analyses [54]– [55]. However, these approaches may be influenced by methodological constraints and the interpretation of their results may be influenced by several variables unrelated to the BBB per se [45, 50, 54, 55]. As these typically elderly patients are exposed to many medications, if such diseases significantly affected brain drug concentrations, it would likely already be known. Even in more pathological conditions such as brain cancer, such as gliomas or metastasis, where the blood-tumor barrier (BTB) is compromised direct assessments have shown that the BTB remains a significant impediment to standard chemotherapeutic delivery and efficacy [56–58].

In other words, the BBB plays a continuous and essential role in maintaining neural function and brain homeostasis; its disruption would result in immediate, severe, and unmistakable physiological and clinical consequences. A non-specific increase in BBB permeability would allow infiltration of the brain parenchyma by monoamines, proteins such albumin and fibrinogen, cytokines, cholesterol, circulating drugs, and various non-electrolytes and electrolyes, among others, disrupting the brain function. Apart from methods that induce BBB dysfunction through hyperosmotic or ultrasound approaches [58, 59], there are very few situations in which increased cerebral bioavailability of drugs can be attributed to an ‘anomaly’ of the BBB. In a clinical setting, a chronic widespread *permeabilization* of the BBB would have obvious consequences that are not observed in ‘real’ life. Therefore, even if weakening of the BBB may, over time, allow larger proteins or cells to enter the brain, such leakage is most often due to subtle, specific changes in cellular function—certainly not the result of a major rupture that would also permit the uncontrolled entry of many small molecules.

**Table:** 

**Recommendations to the study of the blood-brain barrier**
**Recommendation 1**: To truly assess the permeability of the BBB, quantitative methods with smaller compounds (less than 1 kDa) that are specifically blocked by the BBB should be incorporated into studies (e.g. sucrose, inulin).
**Recommendation 2: Avoid terms like BBB ‘disruption’**,** ‘opening’ or ‘breakdown’** but rather use terms like BBB dysfunction or, if relevant, BBB leakage when reporting biological changes in BBB. More broadly, carefully selected and specific terms should be used, avoiding imprecise or ill-defined expressions.
**Recommendation 3**: Always consider the dual nature of the BBB, both as a physicochemical barrier and a dynamic biological interface, when designing and interpreting experiments related to its function and integrity (Fig. 1).

## Conclusion: words matter

In conclusion, the question raised by Dr Stern over a century ago, exposing the dual role of the BBB, still stands to this day. The answer remains that the BBB is both a living biological system and a physicochemical barrier (Fig. 1); recognizing this distinction is essential, as conflating the two perspectives continues to hinder a clear understanding of its role in brain health.

Terminology like “*disruption*”, “*opening*” or “*permeabilization*” is now commonly used to describe changes related to the cellular biology of the BBB. These terms are often misleading as they imply profound changes in the physicochemical properties of the barrier or even hemorrhages. They fail to capture the complexity of the BBB and the wide spectrum of its dysfunctions, which include subtle alterations in barrier integrity, endothelial metabolism, and transporter or receptor function. Their use may also lead to faulty interpretations. It is now common to hear both scientists and the lay public assume that drugs or other blood-borne compounds have easy access to the brain in diseases such as AD or even with age. As such, the myth of the BBB breakdown oversimplifies a complex and dynamic system to a binary state of being, either intact or completely compromised. Understanding the nuanced mechanisms of BBB regulation, the specific contexts in which BBB changes occur, how to assess them experimentally, and the implications for disease progression and treatment are crucial for advancing CNS research and therapeutic development.

## Data Availability

No datasets were generated or analysed during the current study.
